# Cocirculation of Two Lineages of Toscana Virus in Croatia

**DOI:** 10.3389/fpubh.2017.00336

**Published:** 2017-12-12

**Authors:** Nazli Ayhan, Bulent Alten, Vladimir Ivovic, Franjo Martinkovic, Ozge E. Kasap, Yusuf Ozbel, Xavier de Lamballerie, Remi N. Charrel

**Affiliations:** ^1^UMR Emergence des Pathologies Virales (EPV), Aix-Marseille Université, IRD 190, INSERM 1207, École des Hautes Etudes en Santé Publique (EHESP), Marseille, France; ^2^IHU Méditerranée Infection, Assistance Publique Hôpitaux de Marseille, Marseille, France; ^3^VERG Labs, Ecology Division, Faculty of Science, Department of Biology, Hacettepe University, Ankara, Turkey; ^4^Faculty of Mathematics, Natural Sciences and Information Technologies (FAMNIT), University of Primorska, Koper, Slovenia; ^5^Faculty of Veterinary Medicine, Department of Parasitology and Parasitic Diseases with Clinics, University of Zagreb, Zagreb, Croatia; ^6^Medical Faculty, Department of Parasitology, Ege University, Bornova, Turkey

**Keywords:** phlebovirus, Toscana virus, sandfly fever, Sandfly Fever Naples Virus, arbovirus

## Introduction

Toscana virus (TOSV) is a sandfly borne virus (genus *Phlebovirus*, family *Phenuiviridae*) which shows a wide distribution in the Mediterranean basin (1). TOSV was first discovered from *Phlebotomus perniciosus* and *P. perfiliewi* sand flies in 1971 in Italy (2). Since then TOSV has been isolated or detected in France, Spain, Portugal, Morocco, Algeria, Tunisia, Croatia, Greece, Turkey, Cyprus, and Corsica either from sand flies or human samples (3). During the warm season, TOSV is recognized as one of the main causes of aseptic meningitis within endemic countries due to increased vector activity during the warm season. TOSV is the most pathogenic among the phleboviruses transmitted by sand flies; it can affect the central nervous system and cause meningitis and meningoencephalitis (1).

Three distinct lineages of TOSV have been identified so far: A, B, and C, at the outset of this study, only lineage C had been identified in Croatia in the cerebral spinal fluid (CSF) of a patient presenting with meningitis (4). Serological studies demonstrated that TOSV is circulating at a high rate in the islands and along the Adriatic coast of Croatia (5). Serology does not discriminate between the three genetic lineages, the presence of TOSV strains belonging to lineages A and/or B had never been reported. In this study, sand flies collected in Croatia were tested for the presence of TOSV RNA and for subsequent identification of TOSV genetic lineages.

## The Study

A total of 1,453 sand flies were collected from 5 locations in Croatia in July 2015 using modified CO_2_-CDC traps (Table 1). A total of 78 pools, each containing up to 30 insects, were analyzed according to location, sex, and date of trapping (Table 1). They were tested by real-time RT-qPCR for TOSV RNA (6). Two pools, C63 and C64, were positive with respective *C*_t_ values at 22.5 and 35.3. Since sequence analysis of the corresponding PCR product did not allow to identify the lineage, two other PCR assays were used for partial sequencing of the nucleoprotein gene (7, 8). Colinearization of the two sequences obtained from pool C63 resulted in a 576-nt long sequence (GenBank acc no KY867756) (9). From pool C64, only the Sandfly Fever Naples Virus (SFNV) nested PCR was positive and resulted in a 320-nt long sequence (GenBank acc no KY867757) (10).

**Table 1:** Sandfly trapping regions and number of the collected sandflies.

Trapping region	Coordinates (lat./long.)	No. of collected sandflies			No. of pools
		Female	Male	Mix	
Duba	42.60032/18.33946	176	129	30	18
Jesenice	42.59282/18.26899	81	0	25	6
Gornja Ljuta	42.53491/18.39999	22	18	2	4
Zvekovica	42.57636/18.23898	12	9	0	3
Vidonje	42.98244/17.64294	490	55	404	47
Total		781	211	461	78

Virus isolation was attempted by inoculating 50 µL of the homogenate supernatant onto Vero cells as previously described (11). After six blind passages, TOSV was not isolated.

**Figure 1:**
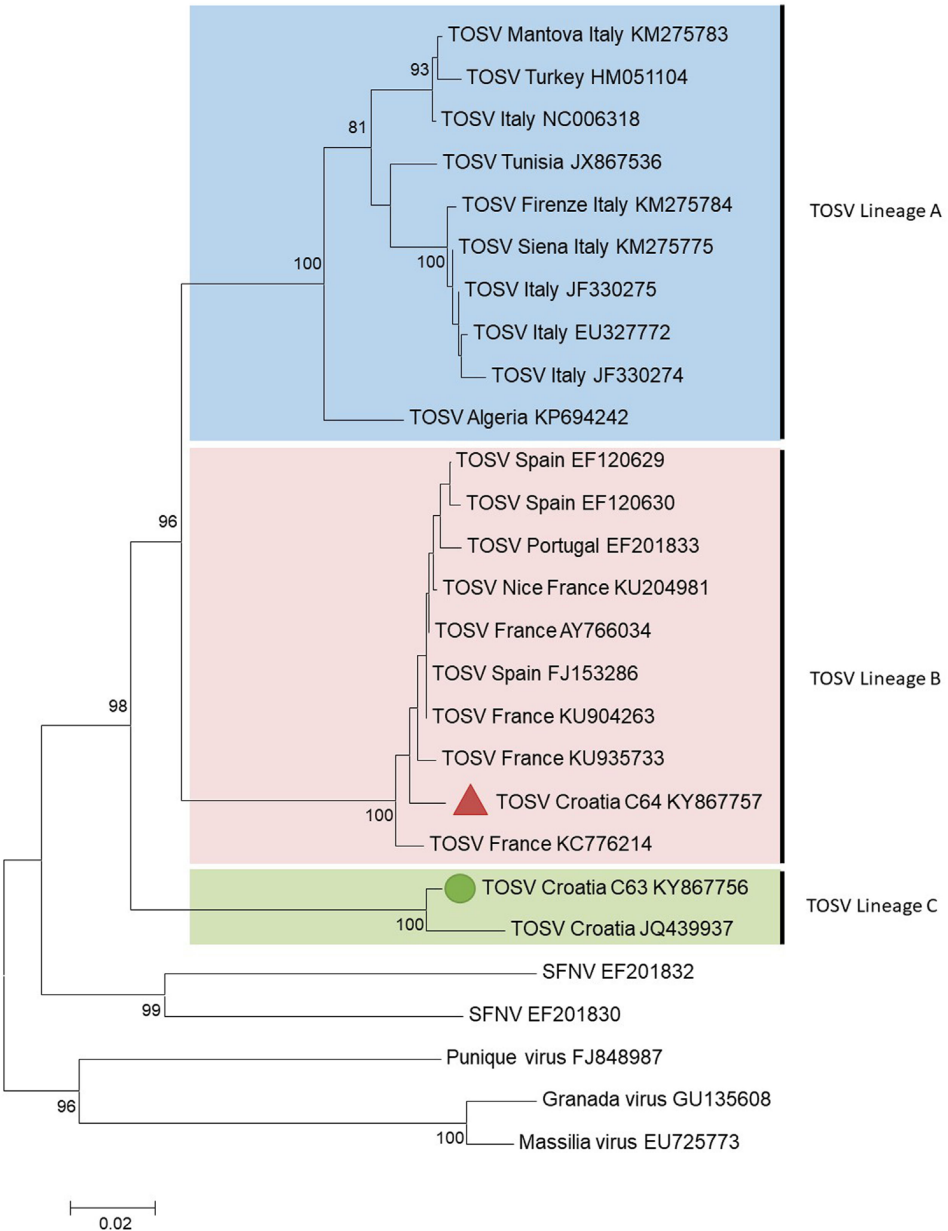
Phylogenetic analysis of Toscana virus (TOSV) based on a 576-nt long sequences between positions 88 and 663 (numbered after strain IssPhL3, Acc No X53794) located in the nucleoprotein gene. Distances and groupings between the N protein sequences were determined by the p-distance algorithm and the neighbor-joining method with the MEGA 6.06 software program (12). Bootstrap values are indicated and correspond to 1,000 pseudoreplications.

C63 and C64 sequences were aligned using CLUSTAL X (MEGA 6.06) with homologous sequences of other TOSV strains and selected phleboviruses belonging to the *Sandfly fever Naples* species obtained from GenBank (see Supplementary Material) (12). Amino acid and nucleotide identities were calculated with the p-distance algorithm. Phylogenetic studies were performed using the neighbor-joining method in MEGA6 (Figure 1). The robustness of the nodes was tested by 1,000 bootstrap replications.

The two TOSV sequences were clearly different from each other with 3.8 and 16.5% genetic divergence at amino acid and nucleotide level, respectively. C63 sequence was grouped with the unique Croatian TOSV sequence (corresponding to the CSF sample of a patient presenting with TOSV meningitis in 2008) (4); hence, C63 sequence was identified as belonging to the lineage C. Surprisingly, C64 sequence was most closely related to the sequence of TOSV strain 113/Nice (GenBank acc no KU204981) isolated from sand flies trapped in southeastern France, belonging to the lineage B. To the best of our knowledge, this is the first evidence that TOSV strains belonging to the lineage B are present in Croatia and probably more largely in the Balkan Peninsula.

Sand fly species from the TOSV positive pools were identified based on cytochrome *b* and cytochrome *c* oxidase subunit I as previously described (13). C63 and C64 pools consisted exclusively of *Phlebotomus neglectus* (Table 2). These results are congruent with the results of a larger study which aimed at building a species inventory of sand flies in the Balkans (VectorNet project supported by ECDC/EFSA) (14). Three sand fly species, *P. neglectus, P. tobbi*, and *Sergentomyia minuta*, were identified from exactly the same location with high dominancy of *P. neglectus* (Vidonje, Tables 1 and 2) in 2015 (Alten et al., unpublished data). *P. perniciosus* and *P. perfiliewi* are the most recognized vectors of TOSV. *P. sergenti* and *P. longicuspis* are also suspected TOSV vectors (15). Here, we provide the first evidence of *P. neglectus* as a probable vector of TOSV. This is a very important finding since this species is present at high density in regions where other TOSV vectors are not present, particularly in the Balkan Peninsula, Eastern Europe, and Turkey. Confirmation of *P. neglectus* as a competent vector of TOSV would considerably increase the size of the exposed human populations.

**Table 2:** Toscana virus (TOSV) positive pools information.

Trapping locality	Code of TOSV RNA positive pools	Sandfly species	Gene region	Reads	No. of sandflies	Gender	Collection date	Altitude
Vidonje	C63	*Phlebotomus neglectus*	*Cyt-b*	541	20	Mix	16/07/2015	240
		*P. neglectus*	*COI*	5,603				

Vidonje	C64	*P. neglectus*	*Cyt-b*	512	20	Mix	16/07/2015	240
		*P. neglectus*	*COI*	9,750				

*Cyt-b, cytochrome b; COI, cytochrome c oxidase subunit I*.

Our results demonstrated that the two TOSV lineages can cohabitate sympatrically within the study area showing no exclusion/interference of one virus by one another (Massilia/Toscana, Punique/Utique, Fermo/Toscana) (16, 17). The same vector species can transmit different types of viruses in the same area. *P. neglectus* is the vector of Corfou virus (18), Balkan virus (19), and TOSV in the same region (this study). Whether a single insect can be coinfected by two viruses is not proven, but is likely to happen and this could drive to the production of a recombinant or reassortant virus (20).

With the assumption that only one insect in each pool is infected, the infection rate of TOSV in Croatia was 0.1% which is higher than rates observed in Tunisia (0.03%), Spain (0.05%), and Algeria (0.004%) (11, 21).

Despite the efforts, virus isolation was not obtained probably because of virion degradation during field collection. Due to the low viral load in pool C64 (*C*_t_ value 35.3) only the SFNV nested PCR was positive and resulted in a 320-nt long sequence. However, the length of the sequence did not affect neither the quality of the alignment nor the topology of the phylogenetic tree.

In Croatia, 37.5% of 755 healthy residents of the coastal regions and islands had TOSV IgG (5). Accordingly, TOSV should be included in the repertoire of pathogens to be explored in patients presenting with neuroinvasive infections during the warm season.

In conclusion, this study showed that (i) strains of TOSV lineage B are present in coastal Croatia where they cocirculate with lineage C strains; (ii) *P. neglectus* is the most probable vector of both lineages of TOSV in the region, (iii) Croatia is, after France and Turkey, the third country where two lineages of TOSV are sympatric; (iv) physicians should consider testing patients who present with neuroinvasive infections in Croatia during the season of sand fly activity for TOSV.

## Availability of Data and Materials

Sequences generated in this study are available in the GenBank database under the accession numbers KY867756 and KY867757.

## Author Contributions

NA performed the sample collection, administered experiments, and wrote the manuscript. BA, VI, FM, OK, and YO organized the sample collection and contributed to the manuscript. XL designed the experiments and contributed to the manuscript. RC designed the experiments and wrote the manuscript.

## Conflict of Interest Statement

The authors declare that the research was conducted in the absence of any commercial or financial relationships that could be construed as a potential conflict of interest.
