# Supplementary material for: Cocirculation of Two Lineages of Toscana Virus in Croatia
Source: Front Public Health. 2017 Dec 12;5:336. doi: 10.3389/fpubh.2017.00336 (PMC5732939; doi:10.3389/fpubh.2017.00336)
Supplement: Supplementary file 1 [file Data_Sheet_1.docx]

**Sequences Used for Phylogenetic Analysis;**

**Toscana Virus Sequences;**

**TOSV Lineage A;**

TOSV Mantova Italy KM275783

TOSV Turkey HM051104

TOSV Italy NC006318

TOSV Tunisia JX867536

TOSV Firenze Italy KM275784

TOSV Siena Italy KM275775

TOSV Italy JF330275

TOSV Italy EU327772

TOSV Italy JF330274

TOSV Algeria KP694242

**TOSV Lineage B;**

TOSV Spain EF120629

TOSV Spain EF120630

TOSV Portugal EF201833

TOSV France AY766034

TOSV Spain FJ153286

TOSV France KU904263

TOSV France KU935733

TOSV France KC776214

**TOSV Lineage C;**

TOSV Croatia JQ439937

**Other Phebovirus Sequences;**

SFNV EF201832

SFNV EF201830

Punique Virus FJ848987

Granada Virus GU135608

Massilia Virus EU725773

>TOSV.Croatia.63

TTTGCTTATCAAGGATTTGACCCAAAGCGCATTGTTCAACTAGTCAAGGAGAGAGGAACTGCCAAGGGCAGAGATTGGAAGAAGGATGTGAAAATGATGATTGTGCTGAACCTTGTTAGAGGGAACAAGCCAGAGGCTATGATGAAGAAGATGTCAGAGAAGGGTGCATCAATTGTCTCCAATCTGATTGCAGTCTACCAGCTGAAGGAAGGAAATCCTGGCAGAGACACCATCACCCTTTCAAGAGTGTCTGCTGCATTTGTCCCTTGGACTATCCAGGCCCTCCGAGTGCTCTCAGGATCTCTGCCAGTGTCTGGGTCCACCATGGATGCTGTTGCTGGAGTGACGTATCCCAGGGCAATGATGCACCCAAGCTTTGCTGGAATCATTGATCTTGACCTGCCAAATGGAGCTGGAGCAACCATTGCTGATGCTCATGGTCTGTTCATGATTGAATTCTCCAAAACAATAAATCCTTCTCTAAGGACTAAACAGGCAAATGAGGTGGCTGCTACTTTTGAAAAGCCCAACATGGCTGCCATGAGTGGTCGATTCTTCACCAGAGAAGACAAGAAG

576nt

>TOSV. Croatia.64

CTGGCAGGGACACCATCACTCTGTCTAGAGTGTCGGCTGCTTTTGTTCCATGGACCGTTCAGGCACTTCGCGTTCTGTCAGAATCGCTGCCTGTTTCTGGGACCACTATGGATGCCATTGCCGGCGTGACTTACCCAAGAGCCATGATGCACCCCAGCTTTGCTGGGATTATTGACCTAGATCTGCCAAATGGAGCTGGGGCCACCATTGCTGATGCTCATGGATTGTTCATGATTGAGTTCTCTAAGACAATAAACCCATCTTTGAGAACTAAGCAGGCTAATGAGGTGGCTGCTACGTTTGAAAAACCCAACATGGCTG

321nt
